# Supplementary material for: WordCommentsAnalyzer: A windows software tool for qualitative research
Source: F1000Res. 2018 Sep 4;7:536. Originally published 2018 May 3. [Version 2] doi: 10.12688/f1000research.14819.2 (PMC6137411; doi:10.12688/f1000research.14819.2)
Supplement: Supplementary file 3 [file f1000research-7-17614-s0002.tgz › b2a79496-4ada-47c4-9682-a99ce867836d.zip!/Eight Iranian Health Journals/Yafteh _55.docx]

© Yafteh  . This work is licensed under CC BY 4.0 (https://creativecommons.org/licenses/by/4.0/).

Volume & Issue: دوره 7 - شماره 2

Title: بررسی موارد تنگی نای بعد از لوله گذاری به دنبال خودکشی

http://yafte.lums.ac.ir/article-1-1073-fa.html

مقدمه: میزان خودکشی در سالهای اخیر رو به افزایش گذاشته است. ارائه یک مجموعه از بیمارانی که بعد از اقدام به خودکشی زیر ونتیلاتور قرار گرفته‌اند و در اثر لوله‌های داخل نای دچار عارضه تنگی در مجرای هوایی (تراشه) شده‌اند، باعث شناخت بیشتر این عارضه و راههای مقابله و پیشگیری از آن می‌شود. مواد و روش ها: طی یک مطالعه آینده نگر، از میان بیمارانی که به علت تنگی مجرای هوایی ناشی از لوله گذاری به مرکز ما معرفی شده بودند، آنهایی که علت لوله گذاری اقدام به خودکشی کرده بودند، بررسی شدند. این کار با پرکردن پرسشنامه تنظیم شده ثبت کامپیوتری اطلاعات آماری و پیگیری منظم بالینی، در مدت 15 ماه (اردیبهشت 1382 تا تیر ماه 1383) انجام گرفت. نتیجه گیری: به‌نظر می‌رسد در بیماران ما مشکلات اجتماعی نظیر بیکاری، تجرد و کم‌سوادی بیشتر از اختلالات روانی زمینه‌ساز اقدام به خودکشی بوده است. بیمارانی که بعد از خودکشی زیر ونتیلاتور قرار می‌گیرند و دچار تنگی تراشه می‌شوند با عمل رزکسیون آناستوموز قابل درمان می‌باشند، اگر چه میزان عود تنگی در آنها بیشتر از گروه‌های دیگر است. یافته ها: از میان 100 بیمار مبتلا به تنگی ناشی از لوله گذاری، تعداد 19 بیمار وارد مطالعه شدند (میانگین سنی 96/9+3/25 سال، محدوده 56-17 سال، 10 نفر مؤنث و 9 نفر مذکر). نوع بیماری و علت خودکشی براساس تشخیص روانپزشک عبارت بود از: 11 مورد استرس روانی اجتماعی در زمینه شخصیت مشکل‌دار یا نابالغ و 7 مورد وجود اختلال روانی، در یک نفر علت نامعلوم بود. علل مستقیم اقدام به خودکشی عبارت بودند از: مشکلات خانوادگی 10 مورد، مسائل عشقی 2 مورد، اعتیاد 3 مورد، ناراحتی سیستم عصبی و افسردگی 6 مورد، مشکلات اجتماعی 2 مورد. بعضی از بیماران 2 علت را ذکر کرده و یک بیمار نیز از ذکر علت خودکشی خودداری کرد. مدت لوله گذاری بیماران به‌طور متوسط 78/14 روز بود (محدوده 3-30 روز)، 8 بیمار تراکئوستومی شده بودند و طول تنگی به‌طور متوسط 12/35 میلی‌متر بود (محدوده 50-20 میلی‌متر). درمانهای انجام شده شامل رزکسیون آناستوموز محل تنگی و نیز دیلاتاسیون متناوب تنگی بود. 3 بیمار با دیلاتاسیون درمان شدند و در 16 بیمار عمل رزکسیون آناستوموز تراشه انجام شد. 8 مورد عود تنگی بعد از رزکسیون ایجاد شد که با رزکسیون مجدد (4 نفر) و با دیلاتاسیون متناوب (2 نفر) بهبودی یافتند و در 2 مورد نیز استنت گذاشته شد. این گروه از بیماران با یک گروه مشابه که به‌علل مختلف دیگری دچار تنگی بعد از لوله‌گذاری شده‌بودند، مقایسه شد. میزان عود تنگی (P=0/011) و طول تنگی (P=0/011) در گروه خودکشی بیشتر از گروه شاهد بود.

Title: بررسی انواع پاتوژن های جدا شده از روپوش کارکنان بیمارستان شهدای عشایر خرم آباد در سال 82-1381

http://yafte.lums.ac.ir/article-1-1074-fa.html

مقدمه: با توجه به اهمیت شناسایی مخازن و منابع احتمالی عفونت در عفونت های بیمارستانی و الگوی حساسیت دارویی آنها مطالعه حاضر انجام شده است . مواد و روش ها: این مطالعه به صورت توصیفی و در سال های 82-81 در بیمارستان شهدای عشایر خرم آباد انجام شد از روپوش کارکنان با احتساب سه مرتبه نمونه برداری جمعا 1020 نمونه گرفته شد. نمونه ها در محیط تایوگلیکولات براث به آزمایشگاه حمل و در آنجا روی محیط های تریپتیکیس سوی آگار یا برین هارت اینفیوژن آگار، مک کانکی آگار و سابورودکستروز آگار ایزوله شدند و کلنی های به دست آمده پس از تعیین هویت به روش کربی-بوئر آنتی بیوگرام شدند و نتایج حاصل با نرم افزار SPSS 7.5 تحلیل گردید . نتیجه گیری: در مجموع در بین باکتری های جدا شده، گروه شایع تر باکتری ها به ترتیب: اسینتوباکترها،کورینه باکتریوم ها، انتروباکتریاسه ها، باسیلوس ها و سودوموناس‌ها بودند و نیز نتایج حاصل از آزمایش آنتی بیوگرام حاکی از حساسیت نسبتا خوب به داروهای آمینوگلیکوزیدی و مقاومت نسبتا شایع در برابر داروهای بتا لاکتام بود. یافته ها: نتایج حاصل از کشت روپوش ها حاکی از آن است که تقریبا 100% نمونه ها حداقل در یک تا سه نوبت نمونه گیری مثبت شدند. بیشترین تعداد سویه های ایزوله شده را گونه های اسینتوباکتر به خود اختصاص داده بودند (یعنی 49/27% کل سویه های ایزوله شده) وآلوده ترین روپوش ها، مربوط به بخش اورژانس و اتاق عمل سرپایی بود.‎

Title: تاثیر آرد جو بر کاهش زردی نوزادان

http://yafte.lums.ac.ir/article-1-1075-fa.html

مقدمه: هیپربیلیروبینمی مشکل شایع نوزادان است که حدود 60% نوزادن ترم ( رسیده ) و 80% نوزادان پره ترم ( نارس) در هفته اول تولد به آن مبتلا می شوند. با توجه به آن که عده نسبتا کثیری از مردم لرستان به طور سنتی از آرد جو برای درمان هیپربیلیروبینمی استفاده می کنند و در موارد بسیاری نوزاد را به پزشک ارجاع نمی دهند , هدف از این مطالعه, آن بود که در صورت موثر بودن آرد جو برای درمان هیپربیلیروبینمی، به عنوان یکی از شیوه های طب سنتی به جامعه پزشکی ارائه گردد و در صورت موثر نبودن به مردم استان اعلام و عوارض ناشی از زردی شدید اخطار داده شود. نتیجه گیری: با توجه به نتایج به دست آمده آرد جو همراه با فتوتراپی مؤثرتر از فتوتراپی به تنهائی بود؛ اما باید به مردم آگاهی داده شود که در صورت ابتلای نوزاد به زردی حتما تحت درمان پزشکی قرار گیرند و آرد جو به تنهائی درمان قابل اعتمادی نیست. یافته ها: از 90 نوزاد مورد مطالعه، 62 نفر(9/68%) پسر و 28 نفر(1/31%) دختر بودند. مدت زمان بستری در گروه مورد در اکثر موارد ( 4/44%) 2 روز با میانگین 7/2روز و در گروه کنترل (3/33%) 4 روز با میانگین 7/3 روز بود. حداکثر مدت زمان بستری شدن در گروه مورد 4 روز و در گروه شاهد 7 روز بود. بین میانگین مدت زمان بستری دو گروه تفاوت آماری معنی داری وجود داشت ( 001/0< P ). تفاوت آماری معنی داری بین میانگین بیلی روبین در دو گروه آزمایش و کنترل طی 24و48ساعت پس از بستری وجود داشت (05/0p<) و مدت زمان بستری در گروه آزمایش کوتاهتر از گروه کنترل بود. مواد و روش ها: این مطالعه به روش کارآزمائی بالینی کنترل شده روی 90 نوزاد رسیده ( ترم) با سطح بیلیروبین mg/dl15 به بالا طی مدت یکسال( 1383) در بخش نوزادان بیمارستان شهید مدنی خرم آباد وابسته به دانشگاه علوم پزشکی لرستان , انجام شد. نوزادان به ترتیب در دو گروه 45 نفره مورد و شاهد تقسیم شدند. در گروه مورد , علاوه بر فتوتراپی , آرد جو نرم و صاف شده روی تمام بدن نوزاد بجز دور ناف و صورت پاشیده شد. در گروه شاهد , نوزادان فقط تحت درمان با فتوتراپی قرار گرفتند. ابزار جمع آوری اطلاعات پرسشنامه و تست های آزمایشگاهی بود.

Title: طراحی روش اندازه گیری مقادیر رادیکالهای فعال اکسیژن در In Vitro و بررسی اثرات ویتامینهای C‏ و E بر روی واکنش رادیکال زایی

http://yafte.lums.ac.ir/article-1-1076-fa.html

مقدمه: رادیکالهای آزاد و در رأس آنها ترکیبات مشتق از اکسیژن فعال (Reactive Oxygen Species=ROS)، مهمترین عوامل شکل گیری واکنش استرس اکسیداتیو هستند. امروزه آسیب رادیکالی به عنوان عامل مؤثری در بسیاری از بیماریها از جمله آلزایمر، آترواسکلروز و سرطان در نظر گرفته می شود. هدف از این تحقیق، ارائه روشی جدید و سریع، برای اندازه گیری رادیکالهای ROS است. مواد و روش ها: این مطالعه توصیفی ـ تحلیلی میزان رادیکال زایی یونهای فلزات واسطه نظیر آهن، مس و وانادیوم در حضور ترکیبات تیول دار گلوتاتیون و سیستئین (به عنوان مواد احیاکننده) و اکسیژن مولکولی، در محیط Invitro با استفاده از آزمون تیوباربیتوریک اسید مورد سنجش قرار گرفت. برای اندازه گیری مقادیر رادیکالهای آزاد تولیدی، از مولکول قند دزوکسی ریبوز، به عنوان معرف استفاده شد. در اثر حمله رادیکالهای آزاد به دزوکسی ریبوز، تعدادی از ترکیبات آلدئیدی نظیر مالون دی آلدهید، تولید شد. سپس مالون دی آلدهید حاصل، با شناساگری بنام تیوباربیتوریک اسید ترکیب شد و محصول رنگی بدست آمده دارای حداکثر جذب در طول موج 532 نانومتر بود. همچنین در این تحقیق، اثرات دو ویتامین آنتی اکسیدان یعنی C و E بر روی میزان رادیکال زایی یونهای فلزات مورد مطالعه، بررسی شد. نتیجه گیری: این مطالعه مشخص بود که واکنش فلزات واسطه با مواد تیول دار در حضور اکسیژن، منجر به تولید رادیکالهای ROS می شود و ویتامینهای C و E، رادیکال زایی را مهار می کنند. یافته ها: نتایج حاصل نشان داد که وانادیوم در مقایسه با آهن و مس، دارای بیشترین توان رادیکال زایی بود و از بین آهن و مس، بسته به این که محیط شامل سیستئین یا گلوتاتیون بود با یکدیگر تفاوت داشت. همچنین ویتامینهای C و E، بر روی ساخت رادیکال آزاد در هر سه یون مورد استفاده، اثرات مهاری داشتند. بخصوص ویتامین C در غلظت صد میکرومولار، دارای بیشترین تأثیر (83درصد) بر روی کاهش تولید رادیکال بود.

Title: اثر همودینامیک تیوپنتون و میدازولام هنگام القای بیهوشی در بیماران عروق کرونر

http://yafte.lums.ac.ir/article-1-1077-fa.html

مقدمه: القای بیهوشی در بیماران قلبی به علت شرایط بیمار و نیز اثرات قلبی ـ عروقی داروهای هوشبر خطر بیشتری داشته و حفظ ثبات همودینامیک در این بیماران از اهمیت خاصی دارد. به نظر می رسد داروی میدازولام ثبات قلبی ـ عروقی مناسبی را هنگام القای بیهوشی فراهم می کند. این مطالعه به منظور مقایسه اثر همودینامیک میدازولام با داروی هوشبر وریدی تیوپنتون در بیماران قلبی انجام شد. مواد و روش ها: تعداد 60 بیماری که عمل بای پس عروق کرونر داشتند و در کلاس 2و3 (ASA) بیهوشی بودند به طور تصادفی به دو گروه 30 نفری تقسیم شدند. در هر گروه، پیش مداوا با داروها و دوزهای یکسان انجام گرفت؛ سپس درگروه اول تیوپنتون با دوز mg/kg 5 و در گروه دوم میدازولام با دوزmg/kg 25/0 به همراه ساکسینیل کولین (mg/kg 5/1) تجویز و آنگاه لوله گذاری داخل نای انجام شد. ضربان قلب و فشارخون شریانی بیماران در زمان های قبل (پایه) و پس از القای بیهوشی و بلافاصله پس از لوله گذاری 3، 5 و 8 دقیقه پس از لوله گذاری اندازه گیری شد. یافته ها: نتایج نشان داد که تجویز هر دو دارو هنگام القای بیهوشی موجب کاهش فشار خون و تغییرمختصر سرعت ضربان قلب شد، همچنین در زمان لارنگوسکپی و لوله گذاری، فشار خون و ضربان قلب در هر دو گروه افزایش یافت که این تغییرات در گروه میدازولام اندکی کمتر بود. نتیجه گیری: یافته های فوق نشان می دهد که میدازولام اثرات همودینامیکی مشابه تیوپنتون دارد. میدازولام، بنزودیازپین کوتاه اثر، حلال در آب و بدون درد است و التهاب زایی وریدی و شروع اثر نسبتاً سریعی دارد و می توان آن را در بیمارانی که منع مصرف تیوپنتون دارند، جایگزین نمود.

Title: اثرات ضد قارچی اسانس گیاه ساتوریا خوزستانیکا منطقه لرستان به روش invitro

http://yafte.lums.ac.ir/article-1-1078-fa.html

مقدمه: با توجه به محدود بودن داروهای ضدقارچی، عوارض جانبی ناخواسته آنها و ظهورسوشهای مقاوم به دارو، تحقیق و پژوهش برای یافتن داروهای ضد قارچی جدید با عوارض جانبی کمتر ،لازم و ضروری می نماید. در این پژوهش اثرات ضد قارچی اسانس گیاه ساتوریا خوزستانیکا که بومی استان لرستان است برروی ده قارچ کپکی و مخمر مورد ارزیابی قرار گرفت. مواد و روش ها: جهت تهیه اسانس گیاه ساتوریا خوزستانیکا عملیات اسانس گیری با روش تقطیر با آب و دستگاه کلونجر انجام شد. محیط های سابورو دکستروز آگار حاوی غلظتهای گوناگونی از اسانس، برای بررسی در ماتوفیتها و ساپروفیتها تهیه و میانگین قطر رشد کلنی ها، پس از 7 و14 روز اندازه گیری شد. در بررسی مخمر کریپتوکوکوس نئو فورمنس دیسکها با غلظتهای گوناگون تهیه و میانگین قطرهاله عدم رشد پس از 7 و 14 روز اندازه گیری گردید. اطلاعات حاصله بوسیله نرم افزار SPSS مورد تجزیه و تحلیل آماری قرار گرفتند. نتیجه گیری: با توجه به نتایج حاصله، می توان امیدوار بود که اسانس ساتوریا خوزستانیکا را می توان در درمان بسیاری از بیماریهای قارچی«درماتوفیت، ساپروفیت و مخمری» و همچنین به عنوان یک ضد عفونی کننده استفاده نمود. یافته ها: نتایج این بررسی نشان داد که غلظت mg/m12% اسانس مرزه خوزستانی، رشد « تریکو فایتون متناگروفیتس، میکروسپوروم جیپسئوم و اپیدرموفایتون فلوکوزوم» و غلظت mg/m1 6% رشد « آسپرژیلوس فومیگاتوس، آسپرژیلوس فلاووس، فوزاریوم، تریکوفایتون وروکوزوم، تریکوفایتون روبروم، میکروسپوروم کنیس» را صد درصد مهارنمود (05/0p<). در مورد کریپتوکوکوس نئوفورمنس، غلظت های مختلف، درصدهای مختلف مهار رشد را نشان داد (05/0p<)، اما حتی در غلظت ماکزیمم mg/m11000 (اسانس خالص) مهار رشد حدود 62 درصد را نشان داد.

Title: بررسی سطوح لیپوپروتئین در بیماران پره‌اکلامپسی

http://yafte.lums.ac.ir/article-1-1079-fa.html

نتیجه گیری: با توجه به یافته‌های پژوهش، دیس‌لیپیدمی به عنوان یک عامل خطر در فیزیوپاتولوژی بیماری پره‌اکلامپسی مطرح است. مواد و روش ها: این پـژوهش یک بـررسی مورد ـ شاهدی است که در آن زنان در حال زایمان با سن حاملگی 40-37 مراجعه کننده به بخش زایشگاه مرکز آموزشی درمانی عسلیان به دو گروه 40 نفره مورد (افراد پره‌اکلامپتیک) و شاهد (سالم) تقسیم شده و مورد مطالعه قرار گرفتند. از خون واحدهای مورد پژوهش نمونه‌گیری به عمل آمد و میزان کلسترول توتال، تری‌گلیسیرید توتال، لیپوپروتئین با دانسیته کم (LDL) و لیپوپروتئین با دانسیته بالا (HDL) اندازه‌گیری شد. یافته ها: در این پژوهش سطح کلسترول توتال، تری‌گلیسرید توتال و لیپوپروتئین با دانستیه کم (LDL) اختلاف آماری معنی‌داری را بین دو گروه مورد و شاهد نشان داد (001/0P<). در حالی که سطح لیپوپروتئین با دانسیته بالا (HDL) در دو گروه اختلاف معنی‌داری نداشت. مقدمه: پره‌اکلامپسی یکی از شایع‌ترین اختلالات دوران بارداری می‌باشد و به عنوان یکی از علل مهم مرگ و میر مادر و جنین به خصوص در کشورهای در حال توسعه شناخته شده است. علت اصلی پره‌اکلامپسی نامشخص است. یکی از فرضیه‌هایی که در مورد فیزوپاتولوژی پره‌اکلامپسی مطرح است، اختلال در سطح لیپوپروتئین‌ها است. هدف از انجام پژوهش حاضر تعیین سطوح لیپوپروتئین‌ها در افراد پره‌اکلامپتیک و مقایسه آن با افراد سالم باردار است.

Title: رابطه سبک رهبری و مدیریت تعارض در بین مدیران بیمارستان های تحت پوشش دانشگاه علوم پزشکی لرستان

http://yafte.lums.ac.ir/article-1-1080-fa.html

مقدمه: بیمارستان به عنوان مرکز ثقل سیستم بهداشتی، درمانی و یک سیستم باز با فرآیندهای پیچیده و منابع متنوع (کیفی و کمی)، جهت رسیدن به اهداف اصیل خود نیازمند یک شیوه رهبری اثربخش و استراتژی مناسب در حل اختلافها و تعارض‎های سازمانی است. هدف از این مطالعه بررسی رابطه سبک رهبری و مدیریت تعارض در بین مدیران بیمارستان های تحت پوشش دانشگاه علوم پزشکی لرستان در سال 83 بود. مواد و روش ها: این پژوهش از نوع مطالعات توصیفی ـ تحلیلی بود. اطلاعات لازم از طریق سه پرسشنامه (مشخصات فردی, سبک رهبری به روش LPC فیدلر و مدیریت تعارض) و نیز مصاحبه، جمع‎آوری و توسط نرم افزار آماری تجزیه و تحلیل شد. جامعه پژوهش را 177 نفر از مدیران 13 بیمارستان تحت پوشش دانشگاه علوم پزشکی لرستان تشکیل می دادند. یافته ها: یافته‎های پژوهش نشان داد که 2/54 درصد افراد سبک رهبری وظیفه‎گرا, 6/35 درصد افراد سبک رابطه‎گرا و 2/10 درصد افراد سبک رهبری مستقل ـ اجتماعی داشتند. از نظر استراتژیهای مدیریت تعارض به ترتیب, 7/49 درصد استراتژی عدم مقابله, 6/44 درصد کنترلی و 7/5 درصد استراتژی راه حل گرایی داشتند. سبک رهبری افراد با میانگین سابقه کار افراد ارتباط آماری معنی داری داشت(043/0 = ‍p). بین استراتژیهای مدیریت تعارض و جنس ارتباط آماری معنی‎دار وجود داشت (036/0 = p). بین سن و استراتژی کنترلی نیز ارتباط آماری معنی‎دار وجود داشت (01/0 = p). نتیجه گیری: تعیین شیوه رهبری ، مدیریت و روشهای برخورد با اختلافها و تعارض‎ها در بیمارستانها حائز اهمیت است. از آنجا که در این پژوهش تعدادی از عوامل موثر در سبک رهبری و مدیریت تعارض مورد بررسی قرار گرفته است, لذا توصیه می شود که در زمینه تاثیر عوامل دیگر مثل: فرهنگ سازمانی, فرهنگ اجتماعی, نژاد , شخصیت و … در شیوه رهبری افراد تحقیقات جامع‎تری صورت پذیرد.

Title: مقایسه ترتیب جمع آوری سلول در پاپ اسمیر و نتایج سیتولوژی حاصل از آن

http://yafte.lums.ac.ir/article-1-1081-fa.html

مقدمه: سرطان دهانه رحم 12 درصد کل سرطانها در خانم ها را تشکیل می دهد. کاهش وقوع سرطان دهانه رحم در کشورهای صنعتی مربوط به انجام تستهای سیتولوژی روتین است. پژوهش حاضر یک پژوهش توصیفی ـ تحلیلی به منظور بررسی مقایسه ای ترتیب جمع آوری سلول از اگزوسرویکس و آندوسرویکس و نتایج سیتولوژی حاصل از آن است. مواد و روش ها: این تحقیق بر روی 1129 نفر از افراد مراجعه‌کننده به درمانگاه های بهداشت‌ و‌تنظیم خانواده تهران انجام شده است. نمونه‌برداری با سیتوبرس برای دهانه داخلی سرویکس و آیر اسپاچولا برای دهانه خارجی سرویکس انجام شد. در 563 مورد برس اندوسرویکال در ابتدا و سپس آیر اسپاچولا و در 565 مورد دیگر ابتدا اسپاچولا و سپس سیتوبرس، به کار برده شد. اسمیرهای تهیه شده مطابق سیستم بتسدا مورد بررسی واقع شدند. یافته ها: آزمونهای آماری بیانگر آن بود که بین دو گروه از نظر سن، سن ازدواج، نوع روش پیشگیری از حاملگی، تعداد حاملگی های قبلی، تعداد زایمانهای قبلی، اشتغال به کار و اعمال درمانی انجام شده بر روی دهانه رحم اختلاف آماری معناداری وجود نداشته است. در بررسی کیفیت لامها نتایج نشان داد زمانی که نمونه برداری ابتدا از اگزوسرویکس به عمل آید، لامهای کمتری به خون آلوده می شوند (P=0.004) ولی نتایج سیتولوژی بدست آمده اختلاف آماری معناداری را در دو گروه نشان نداد. نتایج اسمیرهایی که در ابتدا از اگزوسرویکس نمونه برداری شده بود، موارد سلولهای سنگفرشی آتی پیک با اهمیت نامشخص مشاهده شده 33 مورد (59 درصد) و در مورد افرادی که نمونه برداری در ابتدا از آندوسرویکس به عمل آمد، 20 مورد (6/3 درصد) گزارش شده بود. اختلاف آماری بین دو گروه مشاهده نشده است. هم چنین نتایج مطالعه مذکور بیانگر آن است که زمانی که نمونه گیری در ابتدا از اگزوسرویکس به عمل آمد در 27 مورد (8/4 درصد) سلولهای سنگفرشی آتی پیک با اهمیت نامشخص مشاهده شد و زمانی که نمونه گیری ابتدا از آندوسرویکس به عمل آمد در 23 مورد (1/4 درصد) Squamous Intraepithelial lesion (SIL) مشاهده شد و از نظر تشخیص ضایعات سرویکال اختلافی بین دو گروه مشاهده نشد.نتیجه گیری: با توجه به تعداد بالاتر SIL گزارش شد، زمانی که نمونه گیری ابتدا از اگزوسرویکس به عمل می‌آید و از آنجائی که افزایش آلوده شدن لام به خون زمانی که نمونه گیری ابتدا از آندوسرویکس به عمل می آید، بیشتر است (004/0p=)، لذا بهتر است نمونه گیری از دهانه رحم به هنگام تهیه پاپ اسمیر ابتدا از اگزوسرویکس انجام شود، چرا که به نظر می رسد در این روش تشخیص ضایعات سلولی سرویکس افزایش خواهد یافت.
